# Insights into the tripartite relationship between cervical cancer, human papillomavirus, and the vaginal microbiome: a mega-analysis

**DOI:** 10.1186/s40246-025-00795-w

**Published:** 2025-08-12

**Authors:** Hannah H. Rashwan, Mohammed H. Ali, Mazen M. Mostafa, Raghda Ramadan, Mohamed Mysara

**Affiliations:** 1 Bioinformatics Group, Centre for Informatics Science (CIS), School of Information Technology and Computer Science (ITCS), Nile University, Giza, 12677 Egypt; 2 School of Biotechnology, Nile University, Giza, 12677 Egypt

**Keywords:** Cervical cancer (CC), Human papillomavirus (HPV), Vaginal microbiome, Metagenome, 16S amplicon sequencing, Mega-analysis

## Abstract

**Background:**

Cervical cancer (CC) is the fourth most prevalent malignancy among women worldwide, where 99.7% of the cases are linked to persistent human papillomavirus (HPV) infections. While emerging evidence suggests a role for vaginal microbiome dysbiosis in HPV-driven CC, the specific microbial alterations and their functional implications remain unclear. However, inconsistencies in identifying specific microbial signatures—largely due to heterogeneous study designs, targeted 16S rRNA regions, and data processing methods—have limited the generalizability of existing findings. To address these challenges, we conducted a standardized mega-analysis using a compositionality-aware approach to ensure consistency and minimize technical bias across studies.

**Results:**

Our mega-analysis consolidates findings from five case–control 16S rRNA ampilicon sequencing studies, encompassing 215 samples. Compared to healthy controls, CC patients exhibited significantly higher alpha diversity (Shannon index, p <0.005) and a shift from a Lactobacillus-dominant to a polymicrobial vaginal microbiome. This microbial dysbiosis was characterized by an increased abundance of Porphyromonadaceae, particularly *Porphyromonas asaccharolytica*, and other anaerobic bacterial species such as* Campylobacter ureolyticus*, *Peptococcus niger*, and *Anaerococcus obesiensis *(FDR <0.05). Functional profiling of the altered microbiome revealed enrichment in pathways associated with chronic inflammation, fatty acid biosynthesis, amino acid metabolism, cellular proliferation, invasion, and metastasis.

**Conclusions:**

This mega-analysis presents the most methodologically homogeneous study to date of CC–associated vaginal microbiome using publicly available 16S datasets. Our findings not only deepen our understanding of microbial influences on CC but also pave the way for novel diagnostic and therapeutic approaches potentially enhancing patient outcomes in CC care. These insights open new avenues for clinical interventions that extend beyond conventional HPV-centric strategies.

**Supplementary Information:**

The online version contains supplementary material available at 10.1186/s40246-025-00795-w.

## Background

Cervical cancer (CC) is a neoplastic disease that predominantly affects women, with an estimated half a million new cases reported worldwide on a yearly basis [1]. It ranks as the fourth most prevalent cancer among women globally and accounts for up to 25% of all female malignancies in underdeveloped countries [2, 3]. Despite public health efforts targeting this disease, CC remains a significant cause of mortality, with 7.3 deaths per 100,000 women reported in 2020 [4, 5]. The diverse causes of this disease include smoking, long-term use of oral contraceptives, genetic predispositions, and above all the persistent infection with human papillomavirus (HPV), which highlights the need for in-depth insights to understand its pathogenesis [6].

HPV is identified as a causative agent in almost 99.7% of CC cases, establishing a critical epidemiological link [7]. The relationship between HPV and CC is exceptionally strong, even more than the well-established link between smoking and lung cancer [8]. Although over 90% of HPV infections are self-limiting and resolve without intervention, there is a persistent risk of reinfection. Crucially, it is the persistent infections that most commonly lead to cancer development [9]. The oncogenic potential of HPV is largely due to the viral oncoproteins E6 and E7, which disrupt key tumour suppressors like p53. This disruption leads to uncontrolled cell proliferation, increased angiogenesis, inhibited apoptosis, and impaired DNA repair, ultimately promoting CC development [10, 11].

Given that cancer is a multifaceted disease, the microbial profiling of the cervicovaginal microenvironment offers notable potential for elucidating the functional interactions between host cells and microbes [12, 13]. A healthy vaginal microbiome is typically dominated by Lactobacillus species, but in women with CC, there is a noticeable shift towards an increase in anaerobic bacteria [14]. This dysbiosis may establish a microenvironment that promotes HPV infections and their progression to cancer by altering the local immune response [14–16]. Therefore, it is imperative to characterize the exact microbial players of the vaginal microbiome and their role in HPV persistence and carcinogenesis.

Numerous studies have examined the vaginal microbiome of CC from a variety of perspectives, where most of them agreed on the depletion of the Lactobacillus taxa in this dysbiotic environment [17]. However, their results have frequently been inconsistent or equivocal when it comes to characterizing the CC vaginal microbiome, where they reported varying abundances of different genera [18–23]. In a meta-analysis conducted by Wu M *et al.*, the authors aimed to identify specific microbial species associated with CC. However, their study did not account for microbial compositionality and lacked consistency in the analysed variable regions [23]. Therefore, efforts to consolidate these findings have been impeded by the lack of a unified pipeline, further contributing to more heterogeneity among the studies, which has made it challenging to draw comprehensive conclusions [24, 25].

To address the limitations observed in previous research, our mega-analysis uniformly implemented a standardized workflow across all samples. This approach ensured consistency and minimized potential biases arising from varied data handling techniques previously employed. Additionally, we have implemented a compositionality-aware approach, accounting for the artefactual changes in the relative abundances of bacterial species within the community. By exclusively incorporating studies that confirmed the presence of CC and aligning the studies to only the V4 region, our study not only enhances the robustness of the findings but also improves the interpretation of functional implications.

## Results

### Systematic search results

**Fig. 1:**
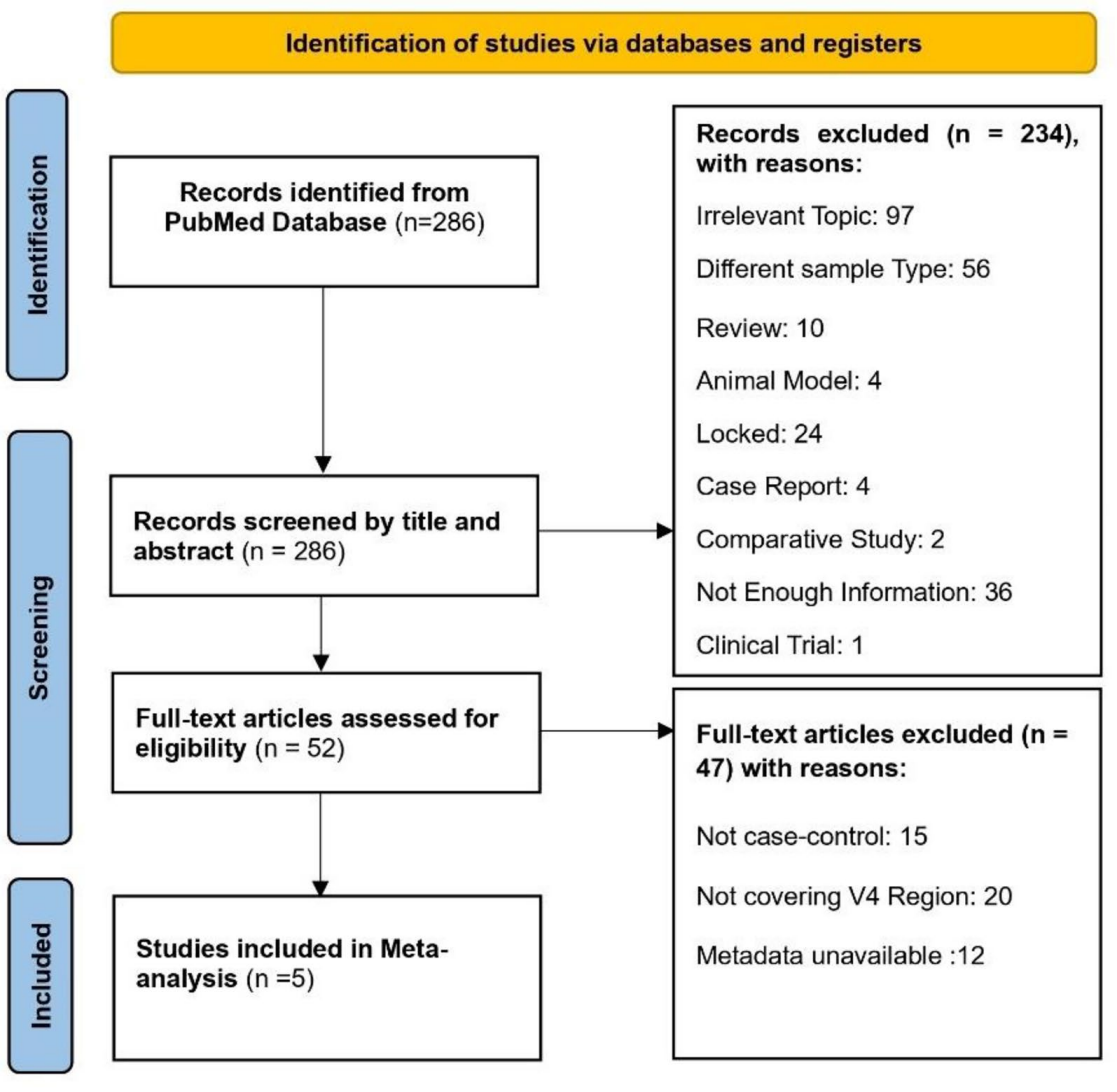
PRISMA chart for Meta Analysis. The chart illustrates the selection process for studies included in the mega-analysis. Initially, 286 records were identified through PubMed database searching. All records were screened, leading to 178 records being excluded for not meeting inclusion criteria. The full texts of 52 studies were assessed for eligibility, out of which 47 were excluded with reasons provided (insufficient data, study design, different v region). Ultimately, 5 studies were included in the analysis. The process adheres to the PRISMA guidelines

A total of 286 records were identified from the PubMed database during the systematic search. 234 entries were excluded for a variety of reasons following the initial screening of titles and abstracts: articles were irrelevant to the subject matter (n = 97), reviews (n = 10), animal models (n = 4), not freely accessible (n = 24), case reports (n = 4), comparative studies (n = 2), lacked sufficient information (n = 36), not vaginal microbiome (n = 56), and clinical trial (n = 1). This resulted in the retention of 52 full-text articles for further eligibility evaluation. 47 articles were excluded from the full-text review due to the following reasons: not a case–control study (n = 15), lack of available metadata (n = 12), and not covering V4 region (n = 20). Ultimately, the mega-analysis comprised five studies (Fig. 1).

In 2021, a study from China utilized 16S rRNA Illumina HiSeq sequencing technology to analyze the influence of pelvic intensity-modulated radiation therapy with concurrent cisplatin-based chemotherapy on the vaginal microbiome, focusing on the V3-V4 variable regions [18]. Similarly, two studies in 2020, one from the United States and another from China, employed the same V3-V4 regions with 16S rRNA Illumina MiSeq sequencing to explore the vaginal microbiome in gynecologic cancer patients pre- and post-radiation therapy [19], and to investigate the relationship between human papillomavirus infection and cervical intraepithelial neoplasia progression [20]. Additionally, studies focusing on the V4 region alone, conducted in the United States in 2019 and China in 2020, used 16S rRNA gene sequencing to decipher the complex interplay between microbiome, HPV, inflammation, and cancer through cervicovaginal metabolic profiling [21], and to reveal the disturbed vaginal microbiome caused by CC [22]. Analysis of HPV status across the included studies revealed a high prevalence of the virus in CC patients. Among the 69 CC patients, 55 (80%) were HPV +. These HPV + patients harbored various high-risk types, including HPV 16, 18, 52, 58, and 59. The analysis comprised 69 CC patients and 146 HCs (Table 1).

**Table 1:** Studies included in the mega-analysis

Country	Numbers	SeqTech &Type	V Region	HPV Status	Age	BMI	Accession no	References
China	6 HC & 20 CC	16S rRNA Illumina HiSeq	V3–V4	18 HPV + (HPV 16,18,52,58,59)	≤ 54: 50%, > 54: 50%	NA	PRJNA687644	[18]
USA	18 HC & 10 CC	16S rRNA Illumina MiSeq	V4	10 HPV +	CC 56.1 ± 13.4y, HC: 59.3 ± 7.8y	CC: 31.4, HC: 28.7	PRJNA518153	[21]
China	68 HC & 9 CC	16S rRNA Illumina MiSeq	V3–V4	9 HPV + (HPV 16/18)	CC: 56.1 ± 9.02y	23.99 ± 0.68 (CC)	SRP122481	[20]
China	25 HC & 18 CC	16 srDNA Illumina Novaseq	V4	18 HPV + (HPV 16,18)	Ctrl–CC, Mean ~ 38.9 y	Mean BMI ~ 30.1	PRJNA595048	[22]
USA	29 HC & 12 CC	16S rRNA Illumina MiSeq	V3–V4	NA	HC: 34.0 ± 3.1, CIN: 35.5 ± 2.9	NA	PRJNA448161	[19]

The table presents an overview of research studies examining the vaginal microbiome of CC. The country where each study was conducted, the number of Healthy controls (HC) and Cervical cancer cases (CC) involved, the sequencing technology (SeqTech) and type used, the accession number for the genomic data, the variable regions (V Region) of the 16S rRNA gene analysed, the type of microbiome studied, the age, BMI and the HPV status are indicated if applicable

### Distinctive microbial composition and increased diversity in cervical cancer compared to healthy controls

**Fig. 2:**
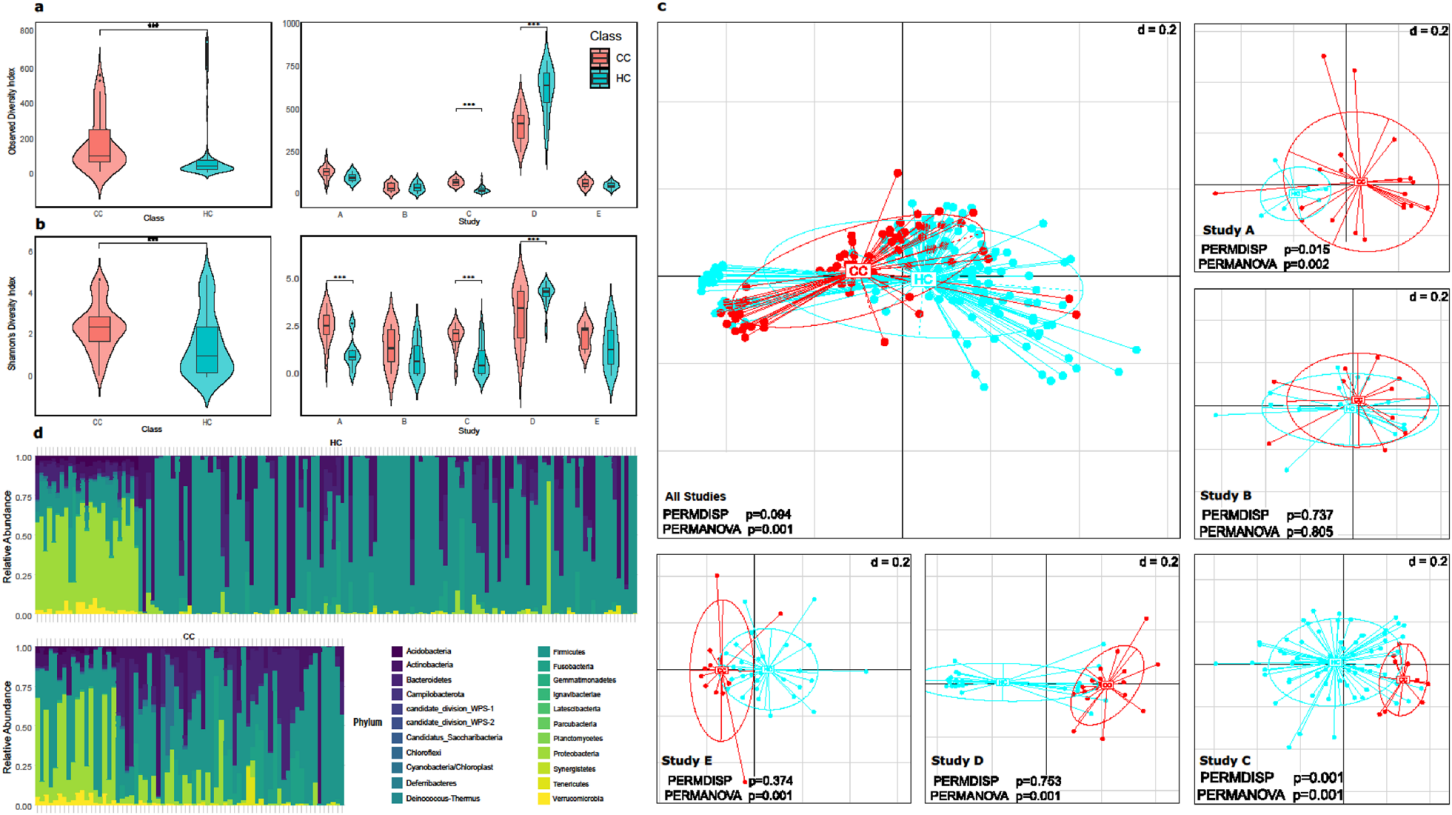
Comparative Analysis of Microbial Diversity and Composition in Cervical Cancer (CC) Patients and Healthy Controls (HC). Violin plots show the distribution of observed microbial richness across CC and HC groups (left) and across individual studies (right). Statistical comparisons were performed using the Mann–Whitney U test; *p < 0.05, **p < 0.01, ***p < 0.001 (**A**). Violin plots represent species diversity across the CC and HC groups (left) and within each individual study (right). Mann–Whitney U test was used for comparisons; *p < 0.05, **p < 0.01, ***p < 0.001 (**B**). The main panel shows beta Diversity (NMDS plots using unweighted UniFrac distances) of all studies combined; side panels display individual study plots. Ellipses indicate 95% confidence intervals. Significant clustering between CC and HC was assessed using PERMANOVA; *p* values are reported (**C**). Stacked Bar Plots of Phylum-Level Taxonomic Composition: Relative abundance of dominant microbial phyla is shown for each sample in the CC and HC groups. Differences in composition reflect the dysbiotic shift associated with CC (**D**)

Our comprehensive examination of the CC and healthy vaginal microbiomes encompassed assessments of alpha diversity, beta diversity, and microbial composition, all of which were derived from five independent studies. The Observed and Shannon indices of alpha diversity demonstrated a significant increase in microbial diversity in CC vaginal microbiome in comparison to HCs (*P* < 0.005; MW; Fig. 2a, b). Upon further examination of the diversity indices for the five investigations A, B, C, D, and E, significant differences in alpha diversities were observed in three of the five studies (A, C, and D) when using Shannon’s Diversity Index (*P* = 0.02–0.001, MW; Fig. 2b). However, when using observed Diversity Index, one study (Study A) was borderline significant (*P* = 0.055; MW; Fig. 2a) and two studies (C and D) were significant out of the five studies (*P* < 0.001; MW; Fig. 2a).

Dimensionality reduction was implemented in the Rhea pipeline to evaluate the beta diversity of all studies that were combined using unweighted UniFrac distances. Nevertheless, the study effect due to the heterogeneities between the studies overshadowed the clear distinction between the CC and HCs groups, although it came out significant (*P* = 0.001; PERMANOVA (Permutational Multivariate Analysis of Variance)). We then analysed the differences within each study individually, and it was in these studies that clusters between the two classes emerged, particularly in studies A, C, D, and E (*P* = 0.001–0.002; PERMANOVA, Fig. 2c). Additionally, we implemented RCM, a more comprehensive methodology, that included all the studies in combination, where it accounts for compositionality and the study as a confounder to elucidate any distinctions between the two classes (*P* = 0.001; PERMANOVA, plots available in Additional file 1). Lastly, bar plots were used to compare the microbial composition at the phylum level between CC samples and HCs. These plots highlighted differences in microbial composition between the cervical microbiome and that of the HCs (Fig. 2d).

### Anaerobic bacterial profiles as potential microbial biomarkers in HPV-positive patients within the cervical cancer microbiome

**Fig. 3:**
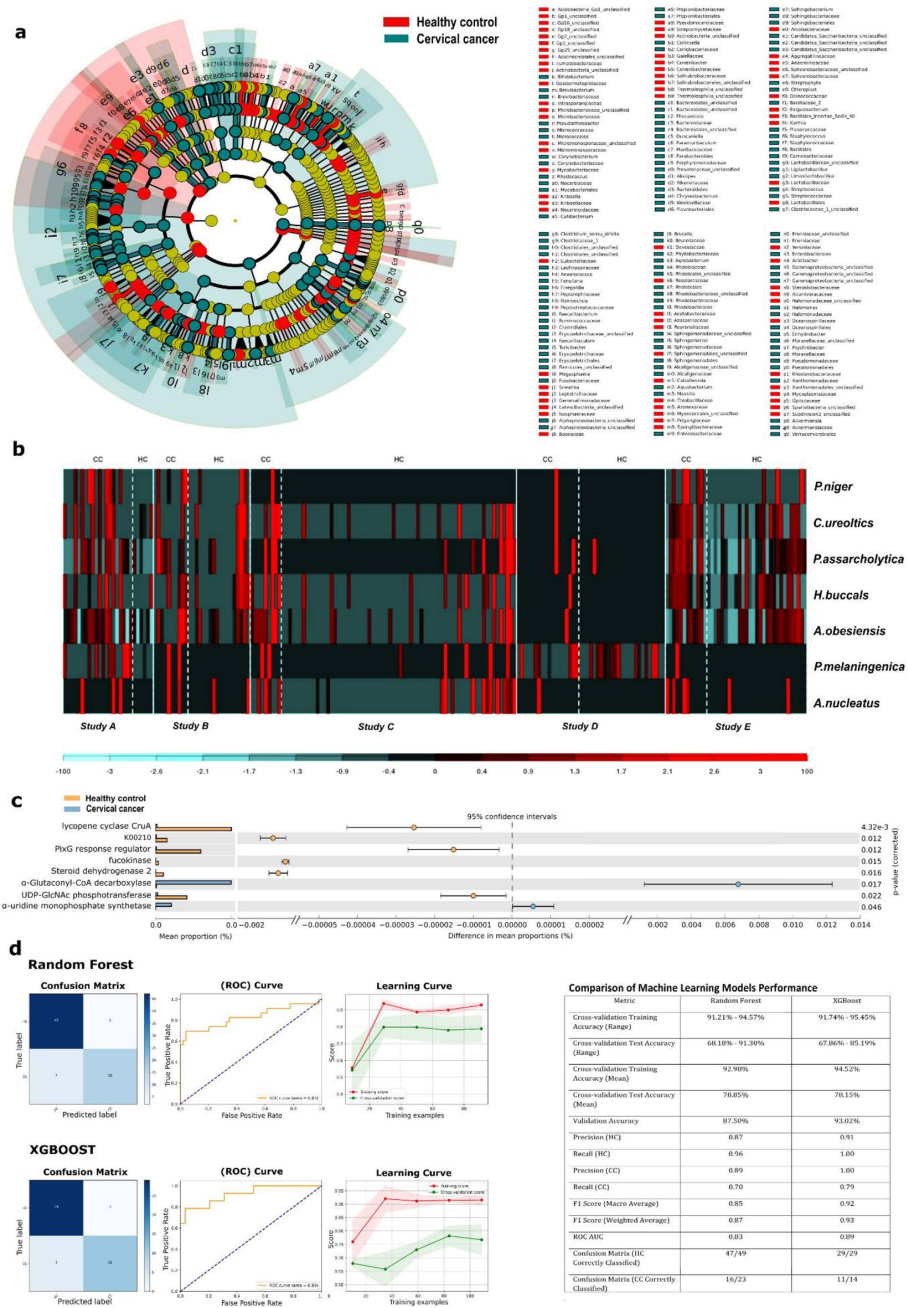
Differential Microbial Signatures, Functional Pathways, and Predictive Modelling in Cervical Cancer (CC) and Healthy Controls (HC). Cladogram depicting significantly enriched taxa at multiple taxonomic levels (phylum to genus). Taxa enriched in CC samples are shown in teal, while those enriched in HC samples are shown in red. Significance was determined using ANCOM-BC (FDR < 0.05) (**A**). Heatmap showing the relative abundance of key microbial species significantly different between CC and HC across included studies, identified by both ANCOM-BC and MetaDE (FDR < 0.05. Red indicates higher abundance, black indicates low or no detection (**B**). Forest plot of KEGG Orthologs (KOs) significantly enriched in CC or HC, based on STAMP analysis. Pathways are ordered by mean proportion difference, with 95% confidence intervals, a broken y-axis was applied to better visualize the scale variation. Significance assessed by Welch’s t-test with FDR correction (**C**). Evaluation of Random Forest and XGBoost machine learning models for classifying CC vs. HC samples. Panels include confusion matrices, ROC curves (AUC_RF = 0.83; AUC_XGB = 0.89), and learning curves showing model stability across training sizes. The accompanying table summarizes performance metrics, including training/testing accuracy, precision, recall, F1-score, and ROC AUC, with XGBoost achieving the highest validation performance (**D**)

A comprehensive analysis was carried out to differentiate between CC and HCs samples at various distinct taxonomic levels. We identified and quantified the observed changes in microbial abundance, with 172 species found to be significantly enriched in CC samples (FDR < 0.05; ANCOM-BC). The results were visualized in a cladogram plot to explain the hierarchical relationships between microbial taxa (Fig. 3a). Many taxa were significantly enriched in CC samples across all six taxonomic levels. Parabacteroides, Staphylococcus, Streptococcus, Anaerococcus, Porphyromonadaceae, and Sphingomonas were the significantly abundant genera in CC compared to HCs (detailed table available in Additional file 1). With in-depth analysis, we were able to identify the differentially abundant species that differentiate CC from HCs. Four species were found to be significantly upregulated in CC (FDR < 0.05) according to both ANCOM-BC and MetaDE analyses namely *Porphyromonas asaccharolytica, Campylobacter ureolyticus, Peptococcus niger, and Anaerococcus obesiensis* (Fig. 3b).

### Microbial signatures associated with HPV-positive cervical cancer

Consolidating data from included studies that reported HPV status, revealed significant differences between the microbiome of HCs and HPV + CC patients. Specifically, alpha diversity was significantly higher in the HPV + cancer samples compared to HCs. Additionally, beta diversity revealed distinct clustering patterns between the two groups, further supporting the presence of significant differences in microbial community structure associated with HPV + CC. We further investigated specific bacterial taxa associated with HPV status from the included studies. Lactobacillus was significantly reduced in HPV + women. Conversely, Sneathia significantly increased in HPV-positive patients, while Prevotella was more abundant in HPV + patients, and increased abundances of Anaerococcus and Dialister were also observed [18, 20–22].

### Functional pathways implicated in carcinogenesis within the microbiomes of HPV-positive patients and Cervical cancer cases

Our functional analysis, conducted using STAMP, identified two Kegg Orthologies (KOs) that were significantly enriched in CC. The enzyme Glutaconyl-CoA decarboxylase subunit alpha was involved in several metabolic pathways, including benzoate degradation and butanoate metabolism. Additionally, GlcNAc-phosphotransferase, crucial in N-Glycan biosynthesis, was also found to be enriched in HC (Fig. 3c).

Further association analysis using MicrobiomeAnalyst highlighted substantial differences in several pathways between CC patients and HCs. Notably, pathways such as fatty acids biosynthesis through several mediators including fatty acid synthase enzyme, amino acids metabolism including altered Valine, Glutamate, Alanine, Aspartate, Tyrosine, Lysine and phenylalanine metabolisms, folate biosynthesis and oxidative phosphorylation, were significantly enriched in CC (FDR < 0.05, detailed list available in Additional file 2).

### Ability of cervical cancer dysbiotic microbiome to distinguish patients from healthy individuals using machine learning models

To investigate the capability of the identified microbial species in differentiating CC patients from healthy individuals, we employed Random Forest and XGBoost machine learning models. The performance of these machine learning models in classifying samples as either HC or CC was evaluated using cross-validation and validation datasets to mitigate overfitting. For the Random Forest classifier, the cross-validation training accuracies were consistently high, with a mean accuracy of 93%. The cross-validation test accuracies, however, showed more variation, resulting in a mean accuracy of 78.9%. Upon validation, the Random Forest model achieved an accuracy of 87.5%, classifying 47 out of 49 HCs samples and 16 out of 23 CC samples, with a precision of 88%. The ROC curve analysis provided an AUC of 0.83, further demonstrating the model’s effectiveness (Fig. 3d).

For the XGBoost classifier, similar cross-validation was conducted with a mean of 94.5%. The test accuracies had a mean accuracy of 78.2%. On the validation set, XGBoost achieved an accuracy of 93.02%, classifying all 29 HCs samples and 11 out of 14 CC samples, with a perfect precision of 100%. The ROC curve analysis provided an AUC of 0.89, further confirming the model's robustness. The learning curve analysis indicated high training scores with a small gap between training and cross-validation scores, signifying a good model fit without overfitting (Fig. 3d).

## Discussion

Despite growing evidence linking vaginal microbiome imbalances to persistent HPV-driven CC, the specific changes in the microbiome and their role remain unclear [26]. This mega-analysis aims to consolidate existing knowledge by elucidating the potential microbial dysbiosis linked to CC and HPV infection. After screening 52 studies focusing on microbial interaction with HPV and CC, five studies were deemed fit for our mega-analysis after rigorous filtering and homogenization. Our results unravelled the microbial composition linked to CC and particularly identified four bacterial species potentially contributing to the persistence of HPV infection and cancer hallmarks.

**Fig. 4:**
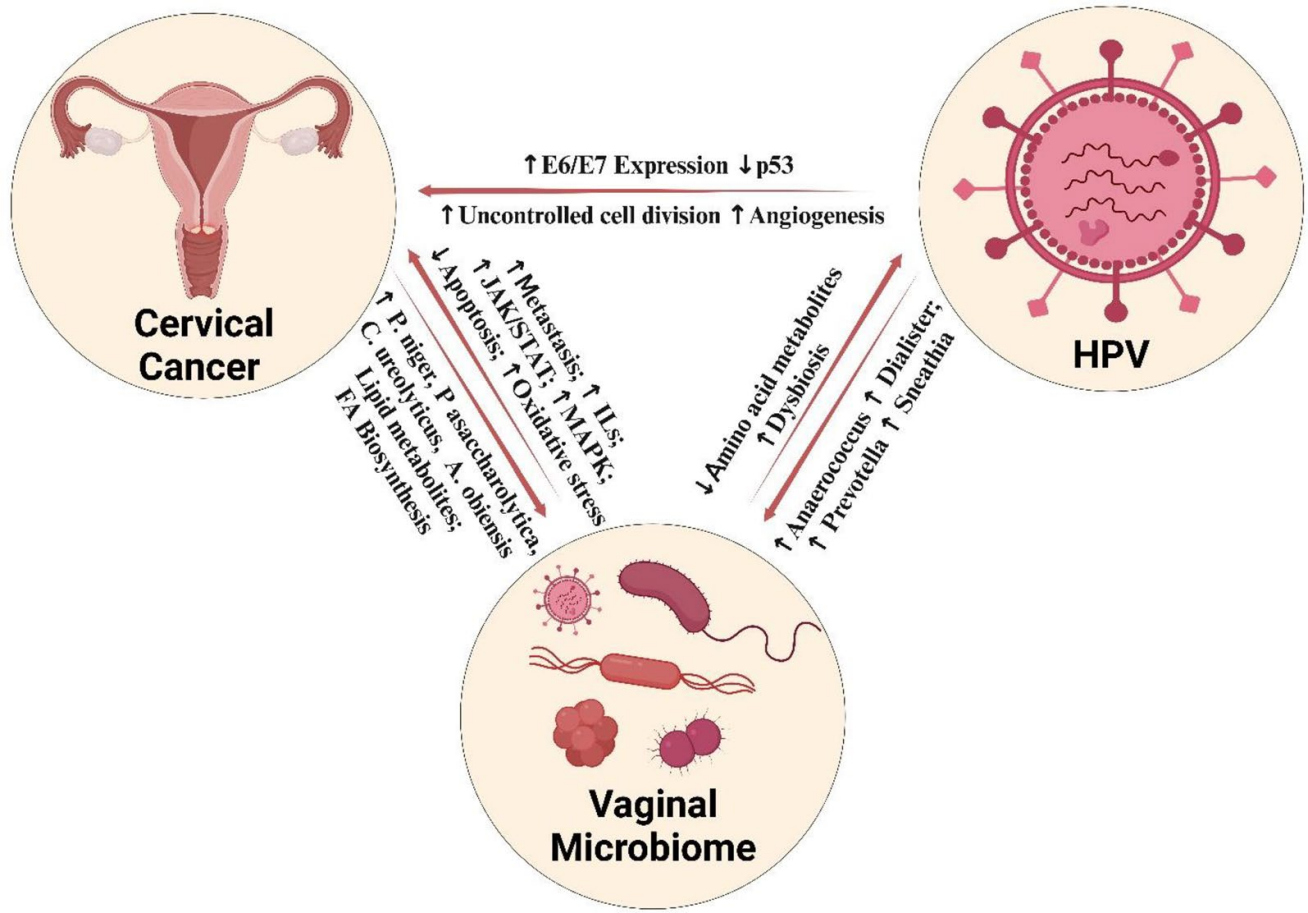
The proposed tripartite relationship between HPV + CC, HPV, and vaginal microbiome showcasing the relatively abundant genera, species and functional pathways and their effect on the progression of the CC and HPV

The tripartite relationship between CC, HPV, and the vaginal microbiome is influenced by several bacterial species and their metabolites (Fig. 4). *Porphyromonas asaccharolytica* was among the most prominent bacteria in CC and HPV patients. With a relative abundance of 1.2%, *P. asaccharolytica* was found significantly enriched across all five studies in CC patients. This bacterium appears to contribute to carcinogenesis through multiple mechanisms. It promotes the expression of proinflammatory cytokines such as IL-6, IL-8, IL-1β, and TNF-α, activates Toll-like receptors (TLRs), and stimulates proliferative signaling pathways including JAK/STAT and MAPK. In parallel, it downregulates proapoptotic proteins and enhances cancer cell migration and invasion—processes that support tumour growth and metastasis [27–29].

Additionally*, P. asaccharolytica* was found to facilitate metastasis through its proteolytic activities by secreting several Matrix Metallopeptidases (MMPs) that disrupt the cervicovaginal niche’s coagulation and extracellular matrix (ECM) [30–35]. These findings align with transcriptomic data from two CC meta-analyses [36, 37], which reported host-level upregulation of arachidonic acid (AA) metabolism—a known driver of MAPK-mediated tumour proliferation [38, 39]. Notably, *P. asaccharolytica* has been linked to AA metabolic activity [40] and elevated production of 12-HETE, a pro-inflammatory lipid mediator [41], suggesting that it may influence host inflammatory tone through lipid signalling. Further supporting its oncogenic potential, *P. asaccharolytica* correlates with the host overexpression of FEN1, a DNA repair enzyme involved in ECM remodelling [36], and with the activation of proliferation- and inflammation-associated transcription factors, including E2F4, ETS1, PCNA, PARP1, and CDK1. Collectively, these interactions underscore *P. asaccharolytica*’s diverse role in promoting a pro-tumorigenic microenvironment, marked by heightened inflammation, suppressed apoptosis, and enhanced metastatic potential.

Beyond *P. asaccharolytica,* our results also revealed other differentially abundant anaerobic microbial species in the vaginal microbiome of CC; namely *Campylobacter ureolyticus, Peptococcus niger*, and *Anaerococcus obesiensis*. Interestingly *A. obesiensis* was reported through this mega-analysis for the first time yet its exact role in CC progression is yet to be determined. It is worth noting that several studies have linked *A. obesiensis* to both prostate and colorectal cancers, suggesting that its oncogenic potential may be mediated through mechanisms involving chronic local inflammation and the release of genotoxic factors [42–45]. Anaerococcus genus -also significant in our analysis- was found predominant in CC, HPV infections and bacterial vaginosis, supporting the potential involvement of *A. obesiensis* in CC [20, 27, 46–50]. Regarding *C. ureolyticus,* it was detected in Cervical Intraepithelial Neoplasia (CIN) lesions as well as HPV patients [51–53]. Additionally, studies focusing on HPV infection further supported our findings, demonstrating a significantly higher abundance of *P. niger* in endocervical samples [51, 54, 55]. Further research is necessary to establish a mechanistic link between these bacteria and CC.

Our analysis also demonstrated significant changes in the overall microbial composition that might influence CC carcinogenesis and progression, alongside HPV infection. We highlighted that CC patients exhibit a higher bacterial diversity with a broader range of microbial species, consistent with other studies [27, 56–58]. Moreover, significant differences in beta diversity were observed in our analysis, suggesting distinct microbial community compositions among these categories, in line with Teka *et al.* [27]. This was characterized by a reduction in Lactobacillales and Lactobacillaceae which are often observed in a healthy vaginal microbiome responsible for maintaining a low pH that acts as a chemical barrier to inhibit pathogenic bacteria and viruses [59, 60]. The shift from a lactobacillus-dominant vaginal microbiome to a polymicrobial one with an increased abundance of anaerobic bacteria is associated with the progression of HPV-related CIN and cancer risk [61–63].

Among the key metabolic pathways enriched in CC, fatty acid biosynthesis was elevated as manifested by the enrichment of fatty acid synthase enzyme; which plays a critical role in several malignant processes such as cancer cell growth, proliferation, migration and invasion [64, 65]. We also observed elevated levels of oxidative phosphorylation, which can be attributed to the uncontrolled proliferation of cancer cells, where they tend to be increasingly reliant on mitochondrial respiration via oxidative phosphorylation [66]. This aligns with the increased production of reactive oxygen species (ROS) reported in another study on HPV + CC tissues [67], suggesting a potential microbial role in promoting oxidative stress, DNA damage, and chronic inflammation [67–69]. Moreover, significant alterations in amino acid metabolism were observed which can indicate a high proliferation rate, consistent with other studies that also reported a disruption in the levels of several amino acids metabolism in CC [21, 70–76]. Notably, these microbial functional shifts align with host transcriptomic signatures involving linoleic and AA metabolism, glutathione metabolism—which is mechanistically linked to oxidative phosphorylation—and pyrimidine metabolism [37, 77]. This convergence between microbial and host pathways points to a potential interplay that may shape the tumour microenvironment and influence CC progression.

While our mega-analysis offers novel insights into the tripartite relationship between CC, HPV, and the vaginal microbiome, certain considerations remain. The inclusion of only five studies ensured methodological consistency but may limit generalizability and introduce study-specific biases. Although we did not incorporate host genomic data, emerging evidence highlights the role of genetic factors—such as loci near PAX8, CDC42, and HLA class II alleles—in modulating immune responses, epithelial integrity, and susceptibility to HPV infection and CC progression [78]. Integrating host genomics with microbial and viral profiles in future studies could enrich our understanding of individual risk and treatment outcomes. The cohorts were primarily from China and the United States, suggesting a need to explore region-specific microbial patterns in more diverse populations. Focusing exclusively on the V4 region promoted consistency across datasets, though minor primer biases may persist. Clinical metadata across studies were limited, with inconsistent reporting of key variables such as age at onset, survival, and treatment response—constraining deeper clinical correlations. Further validation through meta-transcriptomics, metabolomics, and experimental models, including gnotobiotic mice colonized with defined microbial communities, would strengthen the mechanistic interpretation of microbial pathways involved in tumor progression.

## Conclusions

Altogether, our study reveals a significant link between the vaginal microbiome and CC, emphasizing the role of microbial dysbiosis in the disease's development. We were able to identify four bacterial species—P*. niger, P. asaccharolytica, C. ureolyticus, and A. obeisnis*— that may facilitate HPV infection and contribute to carcinogenesis, making them potential diagnostic biomarkers. Although only five studies were included in this mega-analysis, the homogeneity of these studies provides a reliable overview of the tripartite relationship between CC, HPV, and the vaginal microbiome. Moreover, the reliability of these functional predictions could be enhanced by using alternative methodologies, such as shotgun metagenomics. This work not only improves our understanding of the microbial factors in CC but also points out new diagnostic and therapeutic possibilities. However, to fully understand the complexity of these interactions and their broader implications in clinical practice, further comprehensive research is imperative. This should include larger, more diverse study populations to validate the initial findings and deeper molecular investigations to elucidate the mechanistic pathways involved. By advancing knowledge in this area, we can pave the way for more personalized and effective approaches to managing and preventing CC.

## Methodology

### Systematic search

Following the PRISMA (Preferred Reporting Items for Systematic Reviews and Meta-Analyses) guidelines, a detailed search was conducted in July 2023 on PubMed [79, 80]. This search aimed to find datasets using 16S rRNA, utilizing specific keywords such as"CC,""cervical carcinoma,""cervical tumour,""cervical tissue,""cervix cancer,""cervical adenocarcinoma,""gynaecologic cancer,"and"16S,""metagenomic,""metagenomics,""microbiome,"or"microbiota."Inclusion criteria focused on studies collecting vaginal swab samples of CC patients and HCs, particularly analysing V4 region of 16S rRNA, and using Illumina sequencing. We limited our mega-analysis to studies sequenced using Illumina platforms to minimize technical heterogeneity and batch effects inherent to cross-platform integration. This inclusion criterion enhances the comparability of raw data across studies and improves the reliability of differential expression and other transcriptomic analyses [81, 82]. Including data from different sequencing technologies could introduce platform-specific biases that obscure true biological signals. We ensured a comprehensive dataset by including only human studies without geographical restrictions and by requiring that studies provide publicly accessible data and metadata. Studies using shotgun sequencing, involving comorbidities, or entailing antibiotic use were excluded to maintain the integrity of the analysis. A completed PRISMA checklist for the meta-analysis is included in Additional File 3.

### 16S Data pre-processing

Raw 16S sequencing data from five distinct investigations were combined to generate a comprehensive dataset. All of which were retained for analysis. A total of 26 samples were included in Study A, where patients who had received radiation were excluded from the dataset. Additionally, Study B contained 28 samples, while Study C contained 77 samples. A total of 43 samples were incorporated into Study D. Finally, Study E comprised 41 samples where patients who had received radiation were excluded from the dataset as well. Consequently, the final dataset utilized for analysis consisted of 215 samples, with 146 samples HC and 69 samples CC. This was forwarded to the OCToPUS (v.1.0; [83]) pipeline for analysis. Initially, the k-mer frequency approach was employed to individually de-noise the forward and reverse raw readings using SPAdes (v.3.5.0; [84]). Contigs were generated by combining the de-noised paired end reads with Mothur (v.1.39.1; [85]). Unresolved discrepancies between forward and reverse reads or Phred scores < 25 are examples of base ambiguity-containing contigs that were removed. After the remaining contigs were aligned to the SILVA database (v.138.1; [86]) targeting the V4 region, which encompasses locations 14,959–22,588, those that did not align within the reference were eliminated. Additionally, contigs that were exceeding eight homopolymers or had peculiar lengths were eliminated. The trimmed sequence alignment was further refined using the Illumina Paired-End Denoiser (IPED) algorithm (v.1.0; [87]) and CATCh (v.1.0; [88]) was employed for de novo chimera removal. OTU clustering was conducted at a 97% identity level using UPARSE (implemented in USEARCH v.8.1.186; [89]).Subsequently, the default criterion of 80% was employed to conduct the taxonomy classification using the Ribosomal Database Project (RDP) dataset (v.19; [90]). In the analysis, operational taxonomic units (OTUs) that were present in fewer than 10 samples were excluded due to low abundance.

### Diversity analysis

We employed a non-parametric approach for hypothesis testing due to the heterogeneous variances and the data being not normally distributed. In order to compare two groups, we implemented the Mann–Whitney U test and applied a false-discovery rate (FDR) < 0.05 [91].Two indices were employed to quantify alpha diversity: the observed diversity index and the Shannon diversity index [92, 93]. The observed diversity index assesses species richness by accounting for the observed species thereby providing an estimate of the total number of species in a community. Conversely, the Shannon Diversity Index is a diversity metric that evaluates both species richness and evenness.

The microbial communities of various samples were compared, and their similarities and differences were evaluated using beta diversity analysis. First, the analysis employed the Rhea pipeline (v.1.1; [94]), which initially normalized the OTU (Operational Taxonomic Unit) table to guarantee consistency among samples. A distance matrix based on the unweighted UniFrac approach was utilized for the beta diversity calculation. This approach considers the phylogenetic relationships and the presence/absence of taxa. Ward's hierarchical clustering method was implemented to cluster the samples, which enabled the visualization of beta diversity across all sample groups via NMDS plots. In addition, beta diversity was further analysed using RCM (Residuals and Covariance Modelling) to account for compositionality [95]. The significance of the differences between groups was evaluated using PERMANOVA for both NMDS and RCM with a FDR < 0.05 [96].

### Biomarkers detection through mega-analysis

The ANCOMBC (Analysis of Composition of Microbiomes with Bias Correction) method was employed to identify taxa that were differentially abundant across various groups [97]. This method was specifically developed to mitigate biases in microbial compositional data. The precise identification of significant taxa is guaranteed by this advanced statistical method, which also addresses the compositionality and sampling biases that are inherent in microbiome datasets. Taxa were considered significantly differentially abundant if their FDR values were < 0.05.

Furthermore, the MetaDE module of MetaOmics was employed to integrate the individual results from each of the five studies in order to identify the significantly differentially expressed OTUs [98]. A mega-analysis was performed to compare the vaginal microbiome of CC patients with that of HCs. This mega-analysis employed the Random Effects Model (REM) as the normalization procedure, which accounts for differences in sequencing platforms, sample collection protocols, and patient demographics that may induce heterogeneity across the studies [99]. The effect sizes were combined to generate a summary effect size and confidence interval within the REM framework, utilizing appropriate statistical methodologies. In line with the methodology employed by Marzouk *et al.*, differentially expressed OTUs were identified by selecting OTUs with FDR values < 0.05 according to both ANCOM-BC and Meta-DE [100].

### Functional prediction

Metabolic and functional capabilities of the microbial communities in CC and HCs samples were inferred through functional prediction. This analysis employed the *themetagenomics* package and the `*t4f*` function in R [101]. Using the STAMP (Statistical Analysis of Metagenomic Profiles) software, the predicted functional profiles were analysed statistically and visualized [102]. The analysis utilized Welch's t-test (two-sided) with a ratio of proportions filter and an effect size threshold of 10.00 to identify significant functional differences. Utilizing the Kyoto Encyclopaedia of Genes and Genomes (KEGG) database, we conducted a search for applicable metabolic pathways to further clarify the functional functions of the identified microbial communities and association analysis was performed using MicrobiomeAnalyst for pathway elucidation [103, 104].

### Machine learning

Random Forest and XGBoost classifiers were employed in this study owing to their proven robustness, generalization capabilities, and suitability for high-dimensional biological datasets [105, 106]. The Random Forest model was configured with the following optimized hyperparameters: n_estimators was set to 150, max_depth to 10, min_samples_split to 4, and min_samples_leaf to 5. These parameters were identified through an exhaustive grid search strategy (GridSearchCV) to ensure optimal performance. Similarly, the XGBoost classifier was fine-tuned through hyperparameter optimization, targeting key parameters such as learning rate, maximum tree depth, and the number of boosting rounds.

To ensure fair and unbiased model training, a three-stage data splitting protocol was implemented. The entire dataset (N = 215) was first divided into three equal parts. One-third (~ 33.3%) was allocated exclusively for final validation and was not involved in any aspect of model training or tuning. The remaining two-thirds were further partitioned using an 80:20 ratio to derive the training and testing sets, respectively. This resulted in approximately 115 samples for training and 29 for testing. All splitting procedures maintained class balance using stratified sampling.

Model performance was rigorously assessed using stratified fivefold cross-validation on the training data to preserve class distribution and reduce variance. The final evaluation was conducted on the held-out validation set using a comprehensive suite of metrics: accuracy, precision, recall (sensitivity), F1-score, and the area under the Receiver Operating Characteristic (ROC AUC) curve. Accuracy measured the proportion of correctly classified samples, while precision quantified the fraction of true positives among predicted positives [107]. Recall assessed the ability of the model to identify all relevant cases, and the F1-score provided a harmonic mean of precision and recall [108, 109]. The ROC AUC score evaluated the model's ability to distinguish between classes across all thresholds, serving as a threshold-independent performance indicator [110].

## Supplementary Information

Additional file 1. RCM Plots and Taxonomic Enrichment Summary: This document presents RCM plots comparing the microbial community structure between CC and HC samples across five independent studies, along with a combined summary plot. Additionally, Table 1 lists the differentially enriched taxa at phylum, class, order, family, and genus levels.

Additional file 2. Association analysis results. Pathway enrichment results out of MicrobiomeAnalyst.

Additional file 3. PRISMA 2020 checklist for Meta-analysis This file contains the completed PRISMAchecklist used to guide the reporting of the meta-analysis. Each item on the checklist corresponds to a keyP aspect of transparent and standardized meta-analytic reporting, indicating where in the manuscript each criterion has been addressed.

## Data Availability

All data generated or analysed during this study are included in this published article and its supplementary information files.

## Abbreviations

CC: Cervical cancer
HPV: Human papillomavirus
HC: Healthy controls
16S: 16S ribosomal RNA
OTU: Operational taxonomic unit
PRISMA: Preferred reporting items for systematic reviews and meta-analyses
REM: Random effects model
FDR: False discovery rate
NMDS: Non-metric multidimensional scaling
PERMANOVA: Permutational multivariate analysis of variance
ANCOM-BC: Analysis of composition of microbiomes with bias correction
KEGG: Kyoto encyclopedia of genes and genomes
RDP: Ribosomal database project
IPED: Illumina paired-end denoiser
CATCh: Classifier for chimera detection
STAMP: Statistical analysis of metagenomic profiles
AUC: Area under the curve
ROC: Receiver operating characteristic
EC: Extracellular matrix
MMP: Matrix metalloproteinase
TLR: Toll-like receptor
JAK/STAT: Janus kinase/signal transducer and activator of transcription
MAPK: Mitogen-activated protein kinase
NF-κB: Nuclear factor kappa-light-chain-enhancer of activated B cells
IL: Interleukin
TNF-α: Tumor necrosis factor alpha
