# Supplementary material for: Insights into the tripartite relationship between cervical cancer, human papillomavirus, and the vaginal microbiome: a mega-analysis
Source: Hum Genomics. 2025 Aug 12;19:89. doi: 10.1186/s40246-025-00795-w (PMC12341356; doi:10.1186/s40246-025-00795-w)
Supplement: Supplementary file 1 — Additional file 1. RCM Plots and Taxonomic Enrichment Summary: This document presents RCM plots comparing the microbial community structure between CC and HC samples across five independent studies, along with a combined summary plot. Additionally, Table 1 lists the differentially enriched taxa at phylum, class, order, family, and genus levels. [file 40246_2025_795_MOESM1_ESM.docx]

**RCM of CC and HC**

**Study A**

**
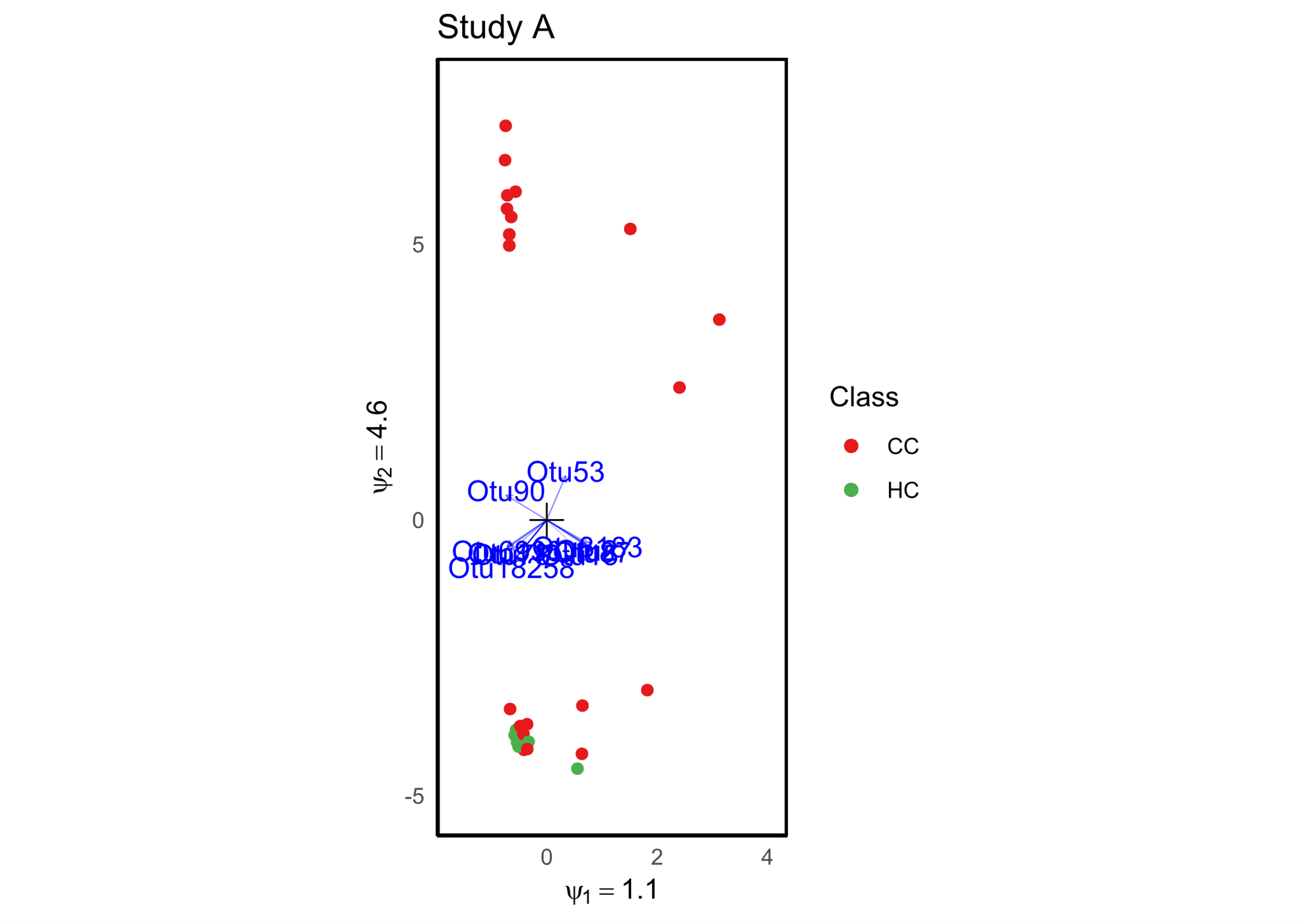
**

**Figure 1:** RCM Plot of Cervical Cancer (CC) and Healthy Control (HC) - Study A.

**
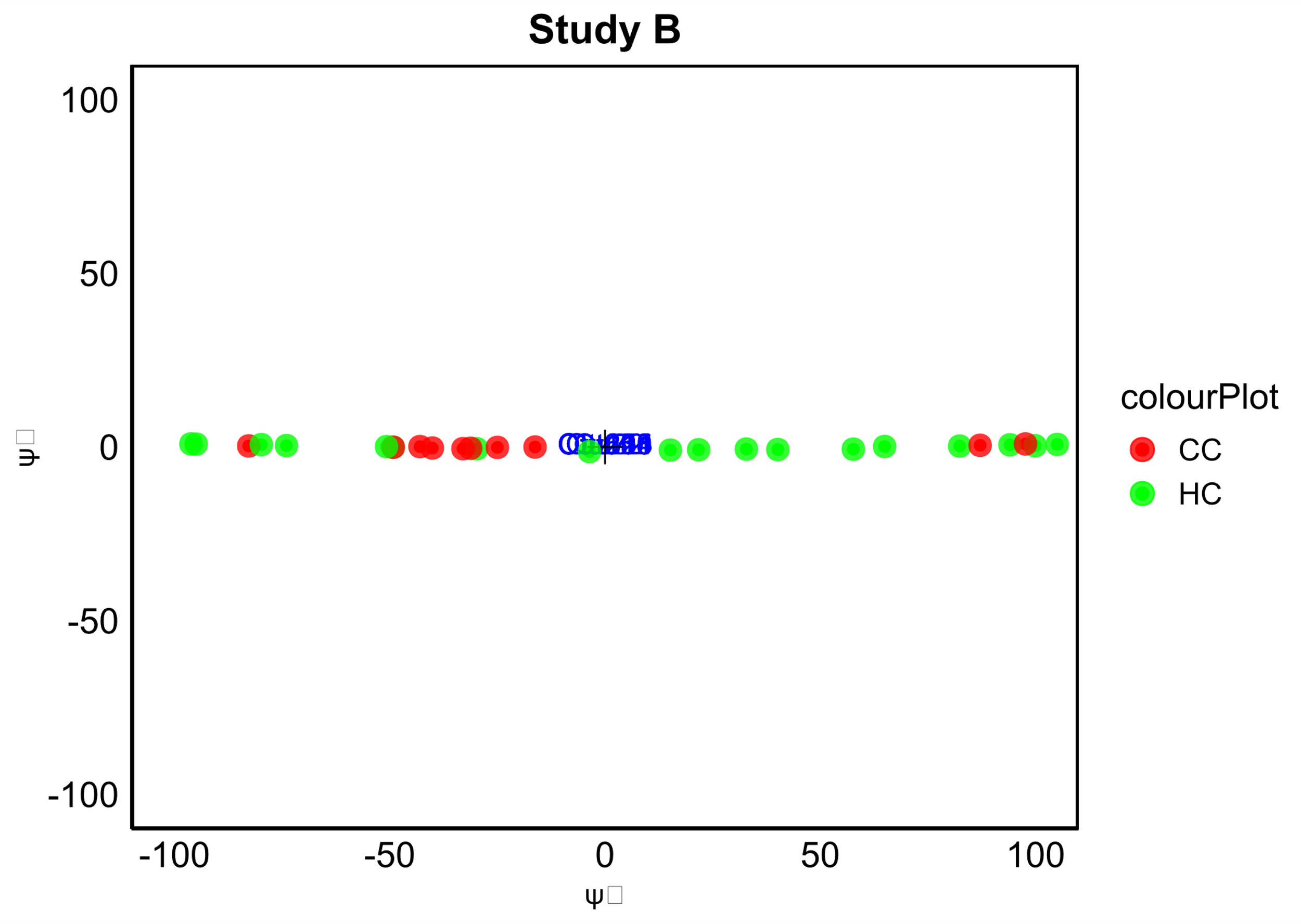
Study B**

**Figure 2** : RCM Plot of Cervical Cancer (CC) and Healthy Control (HC) - Study B.


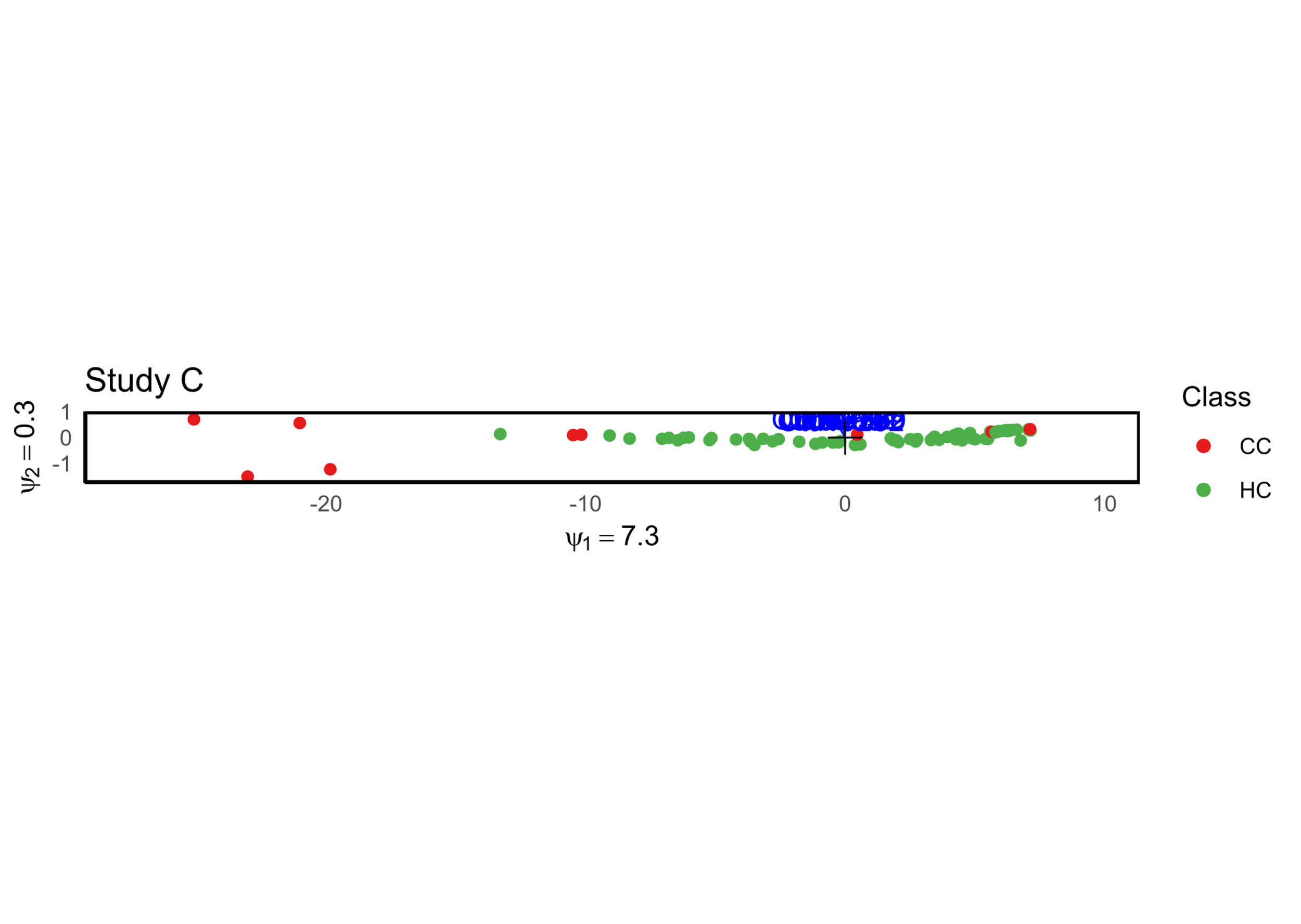
**Study C**

**Figure 3** RCM Plot of Cervical Cancer (CC) and Healthy Control (HC) - Study C.


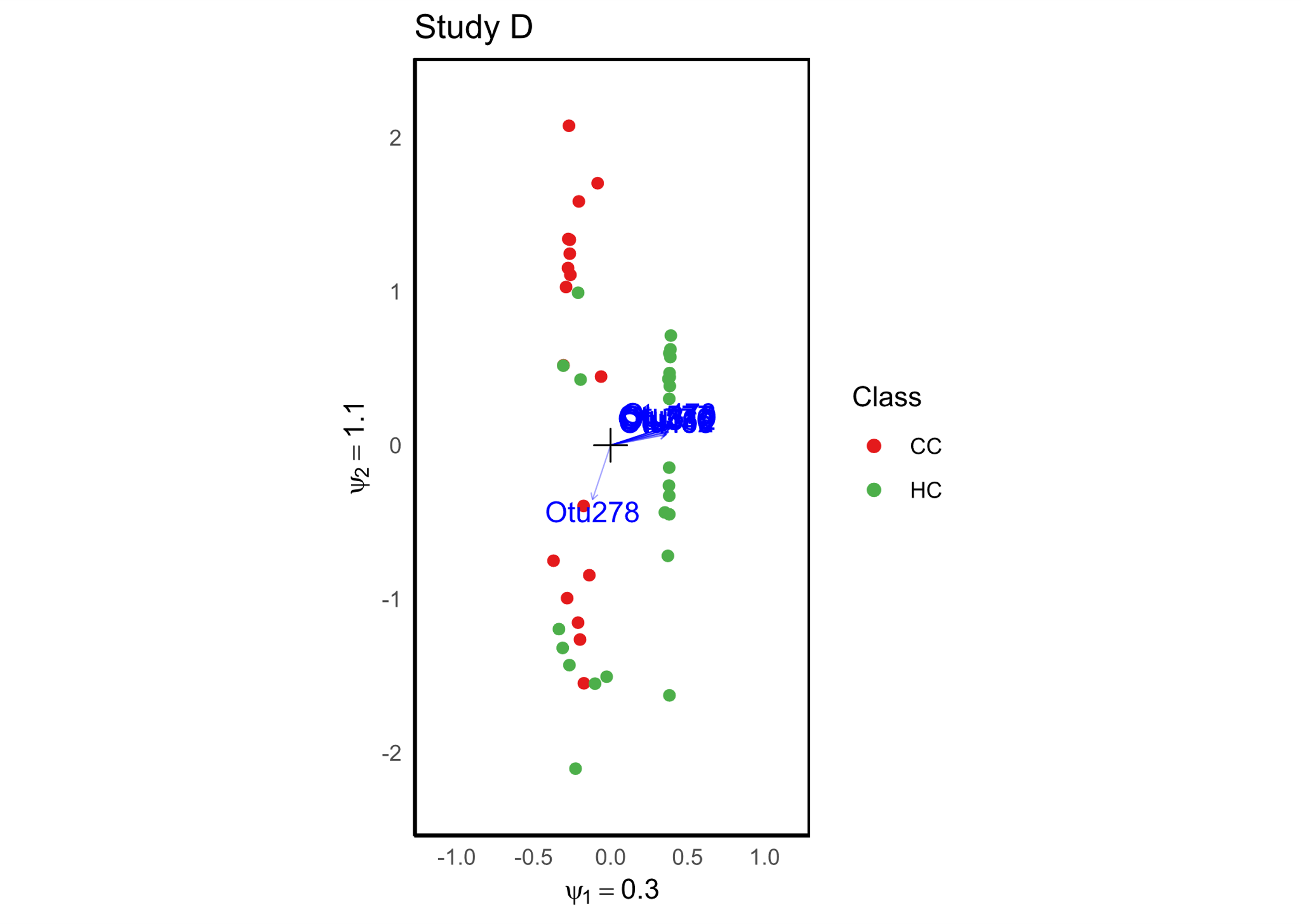
**Study D**

**Figure 4** RCM Plot of Cervical Cancer (CC) and Healthy Control (HC) - Study D.

**Study E**


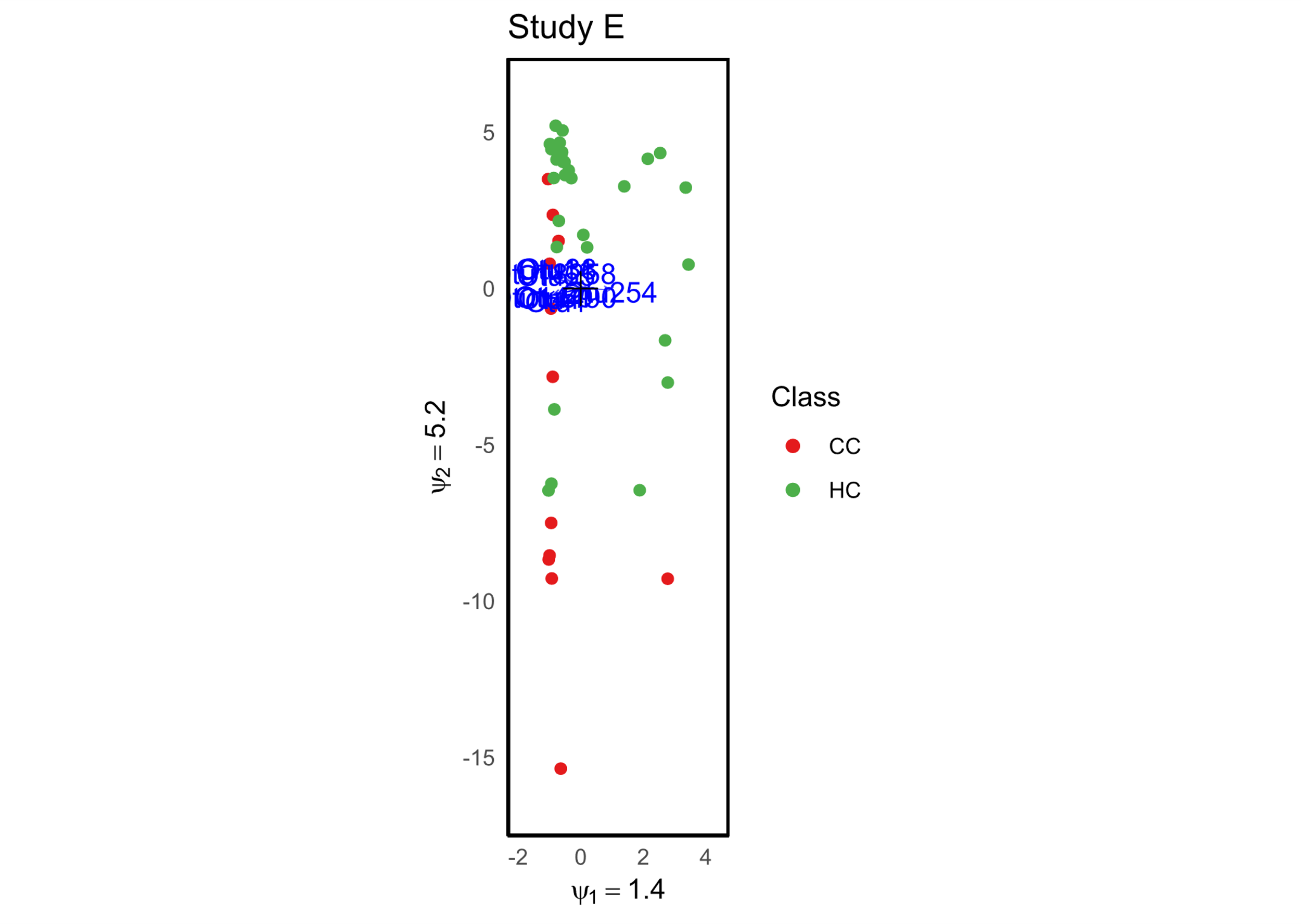


**Figure 5** RCM Plot of Cervical Cancer (CC) and Healthy Control (HC) - Study E.

**All studies**

**
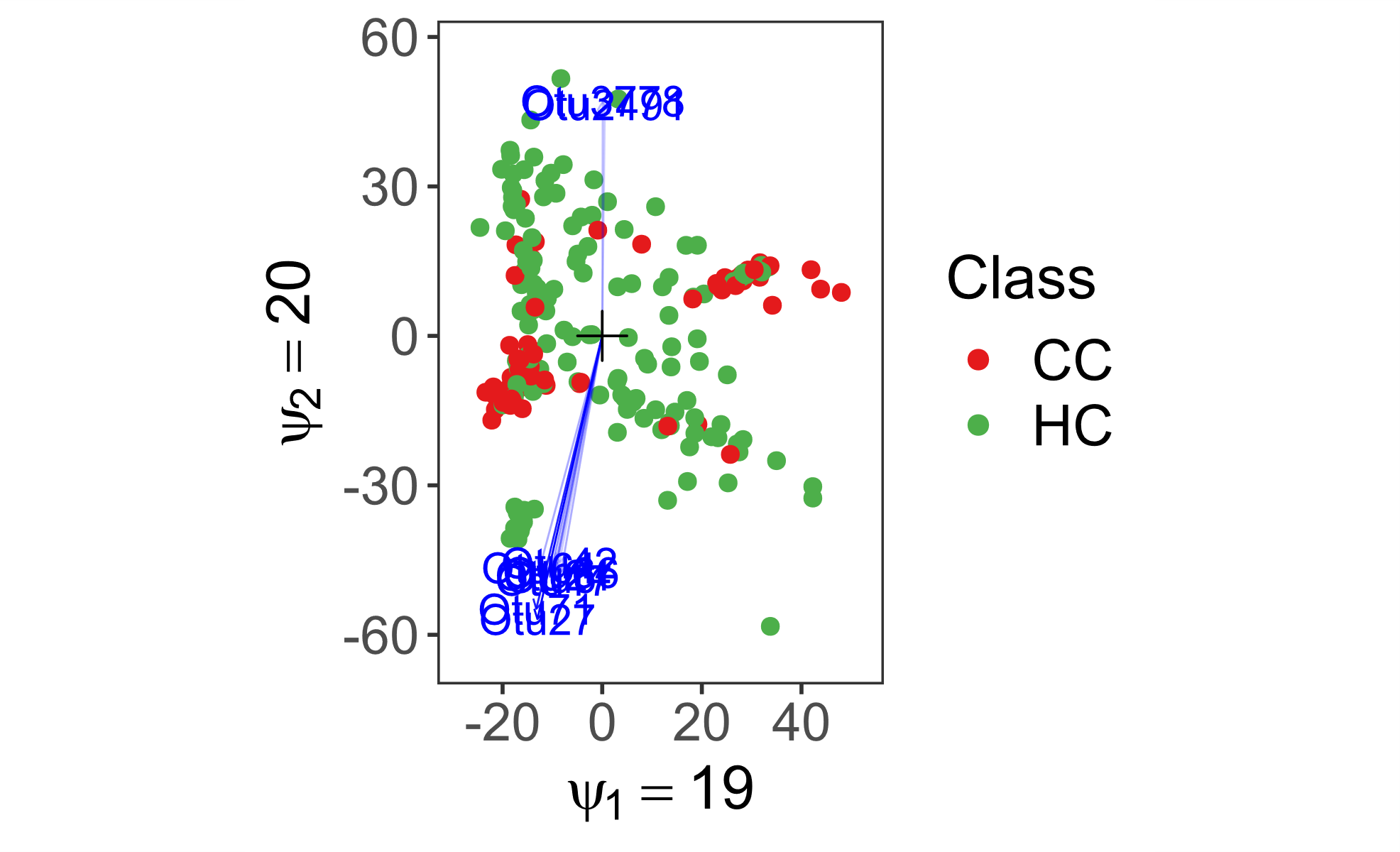
**

**Figure 6** RCM Plot Summary Across All Studies.

**Table 1** Taxonomic Levels of Bacterial Enrichment in Cervical Cancer (CC) and Healthy Control (HC) Samples. The table highlights the enriched taxa across different taxonomic levels in cervical cancer (CC) samples compared to healthy controls (HC).

| **Taxonomic Level** | **CC Enriched** | **HC Enriched** |
| --- | --- | --- |
| Phylum | Sphingobacteriaceae | NA |
| Class | Erysipelotrichaceae, Streptophyta and Chloroplast | NA |
| Order | Micrococcales, Mycobacteriales, Propionibacteriales, Bacteroidales_unclassified, Muribaculaceae, Bacteroidales, Sphingobacteriales, Bacillales, Verrucomicrobiales, Clostridiales, Peptostreptococcaceae, Clostridiales, Erysipelotrichales, Rhizobiales, Rhodobacterales, Sphingomonadales, Alcaligenaceae, Enterobacterales, Gammaproteobacteria_unclassified, Oceanospirillales, and Pseudomonadales | NA |
| Family | Brevibacteriaceae, Micrococcaceae, Corynebacteriaceae, Nocardiaceae, Propionibacteriaceae, Coriobacteriaceae, Bacteroidaceae, Porphyromonadaceae, Rikenellaceae, Weeksellaceae, Sphingobacteriaceae, Bacillaceae, Planococcaceae, Staphylococcaceae, Carnobacteriaceae, Streptococcaceae, Clostridiaceae, Lachnospiraceae, Peptoniphilaceae, Peptostreptococcaceae, Ruminococcaceae, Erysipelotrichaceae, Fusobacteriaceae, Brucellaceae, Phyllobacteriaceae, Rhizobiaceae, Rhodobacteraceae, Sphingomonadaceae, Enterobacteriaceae, Erwiniaceae, Halomonadaceae, Moraxellaceae, Pseudomonadaceae, and Akkermansiaceae | Ilumatobacteraceae, Geodermatophilaceae, Intrasporangiaceae, Microbacteriaceae, Micromonosporaceae, Mycobacteriaceae, Kribbellaceae, Nocardioidaceae, Pseudonocardiaceae, Streptomycetaceae, Gaiellaceae, Conexibacteraceae, Solirubrobacteraceae, Arcobacteraceae, Aggregatilineaceae, Anaerolineaceae, Sphaerobacteraceae, Deinococcaceae, Lactobacillaceae, Eubacteriaceae, Leptotrichiaceae, Gemmatimonadaceae, Isosphaeraceae, Boseaceae, Devosiaceae, Roseiarcaceae, Acetobacteraceae, Azospirillaceae, Reyranellaceae, Caballeronia, Thiobacillaceae, Azonexaceae, Polyangiaceae, Spongibacteraceae, Yersiniaceae, Steroidobacteraceae, Alcanivoracaceae, Oceanospirillaceae, Rhodanobacteraceae, Mycoplasmataceae, and Opitutaceae |
| Genus | Bifidobacterium, Brevibacterium, Pseudarthrobacter, Corynebacterium, Rhodococcus, Cutibacterium, Collinsella, Phocaeicola, Duncaniella, Paramuribaculum, Parabacteroides, Alistipes, Chryseobacterium, Sphingobacterium, Staphylococcus, Ligilactobacillus, Limosilactobacillus, Streptococcus, Clostridium, Anaerococcus, Fenollaria, Finegoldia, Romboutsia, Faecalibacterium, Faecalibaculum, Turicibacter, Brucella, Agrobacterium, Sphingomonas, Aquabacterium, Massilia, Halomonas, Enhydrobacter, Psychrobacter, and Akkermansia. In contrast, HC samples showed significant enrichments of Kribbella, Conexibacter, Exiguobacterium, Kurthia, Megasphaera, Sneathia, Devosiaceae, Caballeronia, and Acidibacter | Kribbella, Conexibacter, Exiguobacterium, Kurthia, Megasphaera, Sneathia, Devosiaceae, Caballeronia, and Acidibacter |
